# Mothers' Perceptions and Attitudes About Infantile Colic in Jazan, Saudi Arabia

**DOI:** 10.7759/cureus.48210

**Published:** 2023-11-03

**Authors:** Mai Mustafa, Talal M Kariri, Rania Q Majrabi, Anwar H Hufaysi, Yumna Abutalib, Raud Khormi, Joud M Alamri, Majidah Halawi, Shaden A Thurwi, Raffan A Alhasani

**Affiliations:** 1 Department of Pediatrics, Faculty of Medicine, Jazan University, Jazan, SAU

**Keywords:** infant, herbs, breastfeeding, soothing techniques, infantile colic

## Abstract

Background

Infantile colic is excessive crying in infants who are otherwise healthy and whose origin is unknown. It is closely linked to maternal depression and is a risk factor for shaken baby syndrome and early cessation of breastfeeding. This study aims to assess mothers' knowledge and attitude about infantile colic in the Jazan region, as well as their knowledge of the causes, and the factors that affect their understanding.

Methodology

This descriptive cross-sectional study was conducted in the Jazan region, focusing on mothers who met the inclusion criteria. The data were collected using a self-administered Arabic electronic survey following participants' consent. The collected data were analyzed using SPSS Version 27 (IBM Corp., Armonk, NY) using the chi-square tests.

Results

This study examined mothers' perspectives on various aspects of infantile colic. A total of 215 (75.43%) out of the 285 participating mothers had experienced colic attacks in their infants, revealing statistically significant differences in urban/rural residence, education, birth order, and breastfeeding practices. Approximately half of the mothers agreed that bottle feeding could lead to colic (49.8%), while around half attributed colic to depression or anxiety (50.9%). Most mothers did not relate breastfeeding (69.8%) or smoking (60%) with colic. Around 55.5% believed that milk protein allergy could be a contributing factor. The majority acknowledged the value of massage (63.5%), placing a warm washcloth (59%), and showering the baby (58.6%). Mothers sought advice from diverse sources (56.5%), often family members (36.5%), with minimal doctor consultation (3.9%). Information about colic management was mixed (48.8%); 27.7% got information solely from healthcare staff, 13.7% exclusively depended on the internet, and 9.8% got information from TV and social media.

Conclusion

This study highlights maternal perspectives and practices regarding infantile colic, showing varying beliefs and approaches that help healthcare providers arrange educational and psychological support to improve infants' and mothers' well-being.

## Introduction

Infantile colic (IC) is considered one of the most common problems in gastroenterology six weeks following birth [1]. It is defined as a "healthy infant with episodes of crying that continue for more than three hours daily, at least three times weekly, and last for at least three weeks during the first four to six months of the infant's life," known as the "3- 3-3 rule." Recent changes have expanded the definition to include healthy newborns who have been irritable and crying for at least one week [2-4].

IC affects around 10% to 30% of newborns during infancy, starts during the second week of life, and then becomes more severe between the fourth and sixth weeks. It usually occurs in the evening without apparent reason and with a normal physical examination, especially in the afternoon, which is characterized by abdominal distension, clenching of the fists, and drawing the legs to the abdomen [2,5,6].

A systematic review study published in 2017 concluded that IC affects 17% to 25% of newborns during their first six weeks of life, and by 8 to 9 weeks and 10 to 12 weeks, these numbers dropped to 11% and 0.6%, respectively [5-7].

IC causes great distress to parents and family members, which has an impact on the physical and mental development of infants, and between 14% and 28% of parents seek medical assistance for this issue [8,9].

The evidence suggests that IC might have several independent causes related to behavioral and physiological theories. The behavioral theory includes insufficient maternal-baby contact, maternal anxiety, and challenging infant temperament, while the physiological theory includes food hypersensitivity, immaturity of gastrointestinal function, and dysmotility [10]. Gastroesophageal reflux disease and cow's milk protein intolerance further contribute to IC [11]. Other risk factors for the severity of colic may include the mother's increasing age and mothers who are smokers or exposed to negative smoking [12].

One of the studies conducted in Turkey concluded that 70.7% of mothers knew about IC; 45% of them received information from health professionals, 18% from the internet and social media, and 13% from family elders [13]. Another two studies conducted in Riyadh, Saudi Arabia, demonstrated a relatively high prevalence of IC with low level of knowledge among Saudi mothers, with 34.4% of their knowledge was principally based on previous similar experiences with siblings, a few of them visiting the doctor, and most of them using anise tea for its treatment [14].

There is no specific medication for IC relief because the disease's pathophysiology is unknown; however, many therapies may be used, including behavioral calming techniques, herbal supplements, and pharmaceuticals. The behavioral hypothesis has inspired strategies including calming the baby with motion and sound, minimizing stimuli, and supportive care, which is an essential item in dealing with IC (hugging, comforting, and distracting baby attention) [2]. Food modification approaches such as encouraging strong adherence to breast milk feeding are essential in reassuring and comforting the baby, as they protect against infant anxiety as breastfeeding can prevent the baby from swallowing air, eliminate protein and lactose, and restrict the use of substances that cause allergies, such as cow's milk. Drinking herbal tea and encouraging nocturnal milk consumption significantly enhance sleep while relieving colic discomfort [15]. Moreover, giving medications such as dicyclomine was found to lessen painful gut contractions and the production of intraluminal gas (simethicone) [15]. Baby massage is also considered an important management technique that is most often used to treat IC as it has been shown to help with skin growth, decrease discomfort, improve the calming and relaxation of the musculoskeletal system, stimulate both the neurological and gastrointestinal circulation of the newborn that may help strengthen bones and muscular stability, lessen stress and anxiety, improve sleep quality, increase weight, lessen crying, and encourage urination [16]. The mother's self-esteem and confidence in her parenting skills are crucial factors in her capability to respond to the infant's needs. Self-efficacy is based on the mother's capacity, culture, attitude, and knowledge to respond to the demands of the infant [2].

This study was conducted to evaluate the attitude of mothers about IC in the Jazan region to assess their knowledge about its causes, symptoms, and factors that affect their knowledge and perspectives. We aim to collect, analyze, and disseminate information to help health professionals educate and guide mothers to better care for their infants with IC.

## Materials and methods

A descriptive, cross-sectional study was conducted in the Jazan region southwest of Saudi Arabia, just north of the Yemeni border. The study included all mothers over 18 years of age in the Jazan region who consented to participate with an infant with IC. The case definition for IC was episodes of infant crying that continue for more than three hours daily, at least three times weekly, and last for at least three weeks during the first six months of the infant's life. It excluded any mother who did not complete the questionnaire or had an unhealthy infant or infant with a chronic disease. The sample size was estimated to be 385 using the Cochran formula, n = (z) 2×p(1-p)/d2, where p = 50% is the anticipated response and z = 95% is the confidence interval, with an error of not more than 5% and a 25% non-response rate. The data were collected via the Arabic language self-administered electronic questionnaire that participants took around 4-5 minutes to complete following their consent to participate.

The data collection started in June 2023 and lasted until August 2023. The questionnaire was prepared by the authors based on the literature and previous studies about IC. It was assessed and revised by experts from the Department of Family and Community Medicine at Jazan University, who offered feedback and suggestions for improvement. The questionnaire was pretested through a pilot study comprising 20 participants to evaluate the time that will be taken to complete the survey and the participant's understanding of the questions. The pilot study data were examined for reliability with a Cronbach alpha of 0.79 but were not included in the main study.

The questionnaire consisted of three parts. The first part was allocated to the demography of the participants, including mothers' and infants' ages, residency, level of mothers' education, methods of infant feeding, order, and number of infants in the family. The second part of the questionnaire assessed maternal perspectives on potential causative factors for IC, including the etiology of colic, infant feeding, and maternal factors (diet, smoking, anxiety). The third part of the questionnaire contained questions related to assessing sources of information and advice about IC and its management and maternal practice regarding IC, including maternal approaches for alleviating colic episodes.

The completed questionnaires were checked to ensure the lack of errors, and all the gathered information was coded into variables analyzed by the SPSS Version 27 (IBM Corp., Armonk, NY) using Pearson's Chi-square test for categorical data. A p-value of less than 0.05 was considered statistically significant.

Ethical approval was obtained from the Institutional Review Board (IRB) of Jazan University (reference number: REC-44/10/637 dated May 3, 2023).

## Results

A total of 285 mothers in Jazan, Saudi Arabia, with a response rate of 74% from the calculated sample of 385, were assessed for their knowledge and attitudes regarding IC, which includes understanding its causes, symptomatology, and methods of calming infants with colic. As shown in Table 1, 75.43% of the mothers who participated in the study had previously experienced colic attacks in their infants, and nearly 60% were from the village. Around 70% of the mothers obtained a university education (holding a bachelor's or postgraduate degree), while almost 50% were employees, 42.1% were housewives, and the remainder were students. Infant feeding was mainly by breast and formula milk in 61.8%, while 11.9% were fed by breastfeeding alone.

**Table 1:** Demographic characteristics of mothers having children with infantile colic

Variable		All n (%)	Have you had a child with infantile colic? n (%)		t/χ^2^	p-Value
			No (n= 70)	Yes (n= 215)		
Place of residence	Jizan city	88 (30.9)	29 (41.4)	59 (27.4)	2.21	0.028
	Jizan villages	179 (69.1)	41 (58.6)	156 (72.6)		
Mother’s education	Primary school	19 (6.7)	6 (8.6)	13 (6.0)	11.455	0.022
	Intermediate school	24 (8.4)	9 (12.8)	15 (6.9)		
	High school	45 (15.8)	11 (15.8)	34 (15.8)		
	Bachelor’s/postgraduate	197 (69.1)	44 (62.8)	153 (71.3)		
Mother’s work	Employee	137 (48.1)	27 (38.5)	110 (51.2)	29.603	0.001
	Housewife	120 (42.1)	28 (40.0)	92 (42.8)		
	Student	28 (9.8)	15 (21.5)	13 (6.0)		
Order of the child	First	58 (20.4)	33 (47.1)	25 (11.6)	68.852	0.001
	Second	66 (23.2)	16 (22.8)	50 (23.2)		
	Third	65 (22.8)	10 (14.3)	55 (25.5)		
	Fourth or more	96 (33.7)	11 (15.8)	85 (39.7)		
Pregnancy term	37-41 weeks	221 (77.5)	55 (78.6)	166 (77.2)	0.416	0.812
	<37 weeks	34 (11.9)	9 (12.8)	25 (11.6)		
	>41 weeks	30 (10.5)	6 (8.6)	24 (11.2)		
Breastfeeding of the child	Yes	208 (73.0)	41 (58.6)	167 (77.7)	10.988	0.004
	Sometimes	45 (15.8)	19 (27.1)	26 (12.1)		
	No	32 (11.2)	10 (14.3	22 (10.2)		
Breastfeeding type	Breastfeeding	34 (11.9)	9 (12.8)	25 (11.6)	11.928	0.036
	Formula milk	27 (9.5)	9 (12.8)	18 (8.3)		
	Breastfeeding + formula milk	176 (61.8)	33 (47.1)	143 (66.5)		
	Breastfeeding + other meals	18 (6.3)	9 (12.8)	9 (4.2)		
	Formula milk + other meals	6 (2.1)	2 (2.8)	4 (1.8)		
	Breastfeeding + formula milk + other meals	24 (8.4)	8 (11.7)	16 (7.6)		

As shown in Table 2, nearly half of the mothers considered IC as a normal frequent event in a child's life rather than a pathological condition. Around 53.3% of mothers expressed disagreement regarding their diet as a potential cause for their child's colic episodes. Approximately half of the mothers agreed and the other half disagreed about feeding bottles serving as a cause of IC and acknowledged that their state of depression and anxiety contributed to causing IC. Most mothers (69.8%) did not consider that breastfeeding was a trigger for IC. More than 60% of women ruled out smoking as a factor behind colic attacks, while 55.5% of them believed that milk protein allergy could be one of the factors influencing IC.

**Table 2:** Maternal perspectives on potential causative factors for colic episodes

Variable	Agree, n (%)	Strongly agree, n (%)	Disagree, n (%)	Strongly disagree, n (%)
Dietary regimen of the mother	98 (34.4)	35 (12.3)	20 (7.0)	132 (46.3)
Feeding bottle	90 (31.6)	52 (18.2)	25 (8.8)	118 (41.4)
Breastfeeding	44 (15.5)	42 (14.7)	89 (31.2)	110 (38.6)
Depression and anxiety in the mother	69 (24.2)	76 (26.7)	41 (14.4)	99 (34.7)
Milk protein allergy	95 (33.4)	63 (22.1)	16 (5.6)	111 (38.9)
Smoking	60 (21.1)	49 (17.2)	25 (8.7)	151 (53.0)
Colic is normal, not pathological	87 (30.5)	36 (12.6)	16 (5.6)	146 (51.3)

Table 3 shows that around 60% of mothers agreed that showering the baby and wrapping them in a towel, placing a warm washcloth on the abdomen, swinging, and placing them in a quiet environment effectively managed the IC. More than half of the mothers disagreed with methods such as placing the child in a car, playing music, providing back massages, or playing music as effective approaches for managing colic episodes. In addition, more than half of the mothers believe in using herbal tea and switching to a different type of milk without seeking consultation from a doctor (53.4% and 58.9%, respectively). Around 63.5% of mothers expressed disagreement with the necessity of consulting a doctor before using herbal remedies, and 73.1% rejected seeking medical advice before administering any medications to address their babies' colic episodes.

**Table 3:** Maternal perspectives/approaches on soothing techniques for alleviating colic episodes

Variable	Agree, n (%)	Strongly agree, n (%)	Disagree, n (%)	Strongly disagree, n (%)
Belly massage	95 (33.3)	17 (6.0)	2 (0.7)	171 (60.0)
Baby shower	140 (36.5)	63 (22.1)	27 (9.5)	91 (31.9)
Breastfeeding	98 (34.4)	51 (17.9)	5 (1.8)	131 (45.9)
Putting a warm washcloth on the child’s stomach	91 (31.9)	77 (27.1)	28 (9.8)	89 (31.2)
Taking the child in the car	55 (19.3)	78 (27.4)	80 (28.1)	72 (25.2)
Playing soft, repetitive sounds for the baby	66 (23.2)	72 (25.3)	64 (22.5)	83 (29.1)
Play music	66 (23.2)	76 (26.7)	70 (24.6)	73 (25.5)
Swing	113 (39.6)	49 (17.3)	30 (10.5)	93 (32.6)
Wrap the child in a towel	104 (36.5)	63 (22.1)	27 (9.5)	91 (31.9)
Massage between forefinger and thumb	100 (35.1)	81 (28.4)	32 (11.2)	72 (25.3)
Putting the child in a quiet place	106 (37.2)	55 (19.3)	24 (8.4)	100 (35.1)
Putting the child in a sitting position when feeding	101 (35.4)	40 (14.0)	21 (7.4)	123 (43.2)
Back massage	110 (38.6)	27 (9.5)	8 (2.8)	140 (49.1)
Change of the mother’s food	105 (36.8)	47 (16.5)	5 (5.3)	118 (41.4)
Herbal tea	91 (31.9)	61 (21.5)	48 (16.8)	85 (29.8)
Change milk	114 (40.0)	53 (18.6)	10 (3.5)	108 (37.9)
Do not use herbs without consulting a doctor	81 (28.4)	23 (8.1)	42 (14.7)	139 (48.8)
Do not use drugs without consulting a doctor	70 (24.4)	7 (2.5)	29 (10.2)	179 (62.8)

As elaborated in Table 4, approximately one-third of mothers (30.2%) indicated that they did not employ any herbal remedies for colic episodes. Among those who did use herbs, slightly more than half (50.5%) reported using anise. Concerning the administration of soothing medications, nearly 30% of mothers did not use any medication during their baby's colic episodes, while 37.2% of mothers could not recall the specific name of the medication.

**Table 4:** Frequency of utilized remedies for infantile colic

Remedies	n (%)
If you use herbs to treat colic, which of the following herbs do you use?	
Anise	144 (50.5)
Chamomile tea	25 (8.8)
Cumin	7 (2.5)
Fennel tea	22 (7.7)
Linden	1 (0.4)
I do not use herbs	86 (30.2)
If you use drugs to treat colic, then what kind of drugs do you prefer for your child?	
Probiotics	25 (8.8)
Simethicone	37 (13.0)
Probiotics + simethicone	5 (1.8)
Herbal drops	27 (9.5)
I do not use drugs	85 (29.8)
I do not remember	106 (37.2)

**Figure 1:**
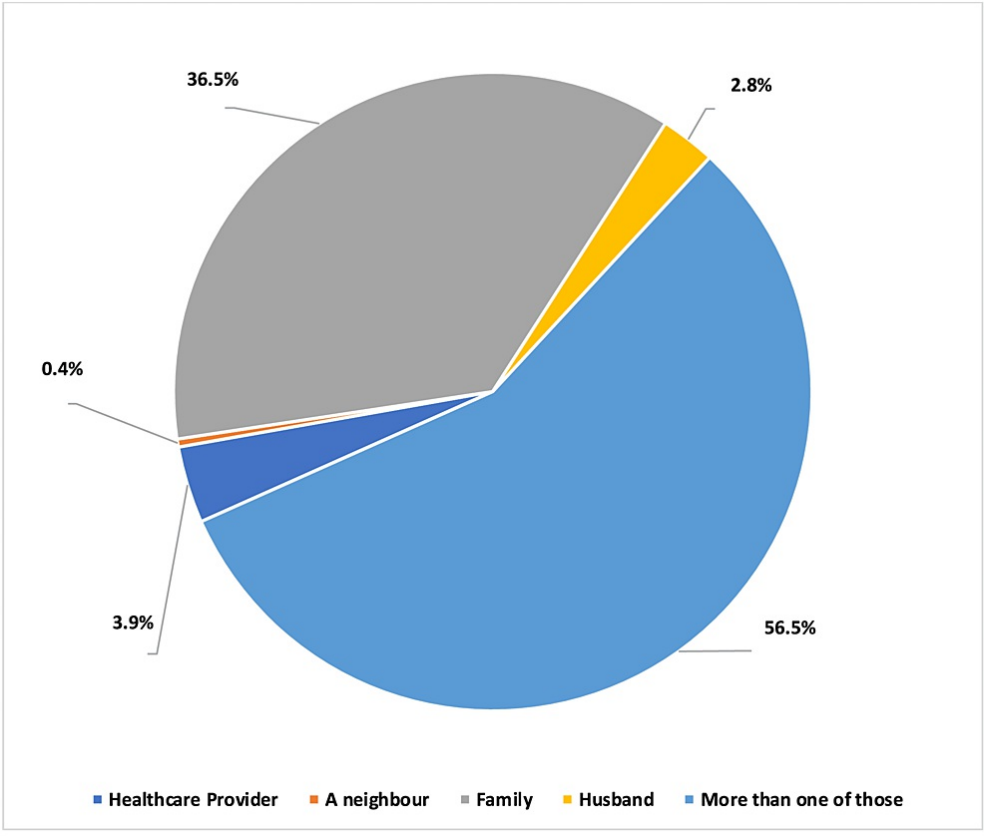
Sources of information for mothers about infantile colic

As shown in Figure 1, only 4% of mothers seek advice and information about IC from a health professional, while most of them (56.5%) sought advice from multiple sources, which included neighbors, family members, and husbands, and 36.5% took their information from a family member.

**Figure 2:**
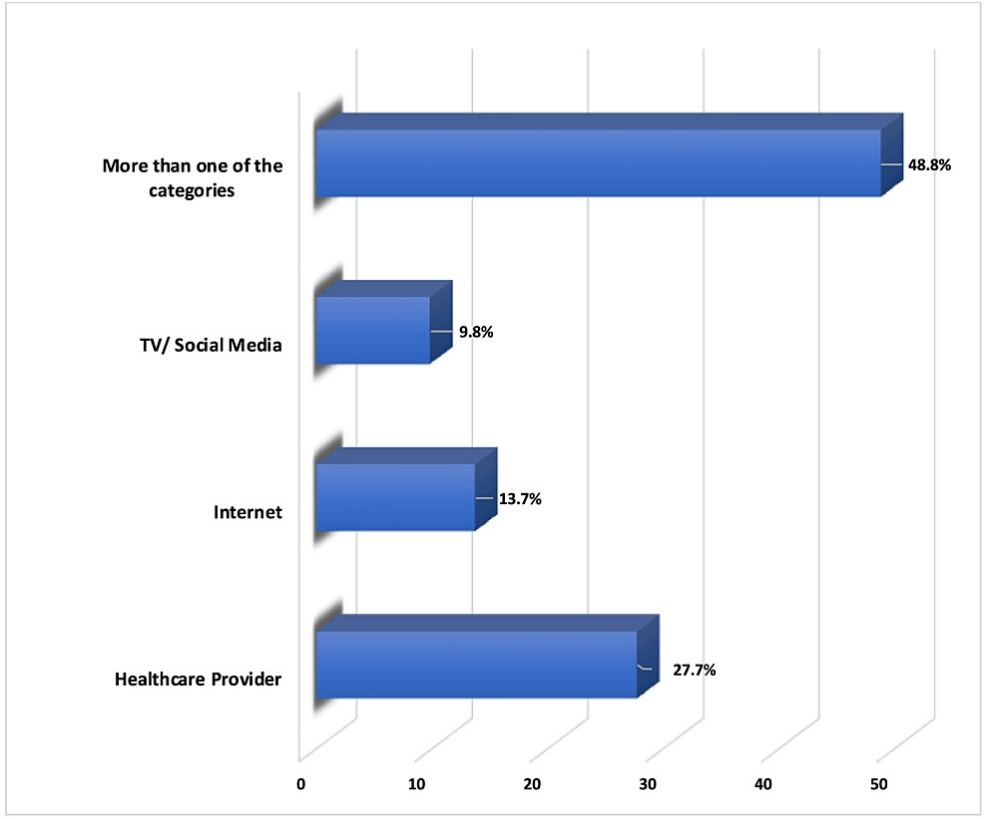
Sources of advice for mothers about the management of infantile colic

Based on the bar chart in Figure 2, most mothers (48.8%) obtained management advice from IC from multiple sources, including healthcare staff, the internet, social media, and TV. In comparison, only 27.7% received it solely from healthcare staff, 13.7% exclusively depended on the internet, and 9.8% relied on TV and social media.

## Discussion

In this study, a considerable proportion (75.4%) of mothers reported having previous knowledge of IC episodes, which is higher than in another Saudi study conducted by Al-Shehri et al. in Riyadh city, where only 20% of women had encountered previous knowledge of IC and this might be attributed to that Jazan is a small city with similar background and culture and that most of their information was gained from mothers, grandmothers, and relatives in contrast to Riyadh, which is the capital of Saudi Arabia [14]. Furthermore, working mothers, those with higher educational levels, and those having more than four children have a better understanding of IC colic pain and its management approaches, as these mothers were better trained to manage colic episodes due to their prior awareness and experience; furthermore, 66.8% of mothers disagree that breastfeeding is a cause for IC, and the majority (69.8%) perceived breastfeeding as beneficial to relieve episodes of IC, which is comparable to a conclusion from a previous study consistent with what was elaborated in Egyptian research led by Safaa et al., highlighting its role in protecting infants against various diseases, including IC [15].

The results of our study support findings from previous studies that there is a link between IC and maternal anxiety and depression [16], as more than half of the mothers (50.9%) acknowledged the potential influence of psychosocial factors on colic occurrence [17]. Approximately 53.3% of the mothers disagreed about the possible link between their dietary intake and the onset of colic in their breastfed infants, in contradiction with a systematic review of randomized controlled trials that concluded a potential therapeutic benefit in adjusting the diet of breastfeeding mothers, involving the elimination of milk products and other types of protein-rich foods [18]. Abdominal massage was relatively of limited value in relieving pain, as reported by the majority of mothers in this study; these findings are consistent with the conclusions drawn by Huhtala et al., where their study indicated that infant massage failed to reflect improvements in colic episodes [19]. Our study findings support using several techniques for alleviating colic discomfort in infants, such as placing a warm washcloth on the baby's abdomen, showering the infant, and wrapping them in a towel, which is similar to the report of other studies, which reveals that swaddling is effective in protecting the infant from heat and cold as well as improving sleep quality and reducing pain [20]. Most of the mothers believed that shaking the baby during colic episodes and providing a calm environment are potential sources of comfort, supporting findings from previous studies [20]; however, our study findings concluded that placing the colicky infant in a car ride failed to alleviate symptoms, reflecting similar conclusions of the study by Roberts et al. [17], while other studies have explored diverse strategies to reduce crying in colicky infants, including using soothing motions, preventing excessive stimulation, employing pacifiers, adopting proactive holding and carrying practices, and using infant carriers [21].

Among the mothers surveyed, more than half held the perspective that seeking medical advice for herbal or medical remedies was unnecessary when dealing with colicky infants, which is similar to a conclusion from another study conducted in Nigeria in 2009, which revealed that 28.1% of mothers sought information about IC from physicians, while a significant majority of 71.9% preferred home-based interventions [22].

Our study also concludes that approximately 53.4% of the participants agreed that using herbal tea could alleviate colic symptoms effectively, which is similar to the findings of a previous study by Weizman et al., which concluded that administering herbal tea three times a day at a dose of 150 mL per dose successfully reduced crying among colicky infants [23], while a different study reported that 33% of mothers do not use any management techniques for IC, 27.4% of them prefer the use of herbal medicines, 18.3% considered alteration of milk formula as a remedy, and 1.7% reported that they would stop breastfeeding and start formula feeding [8]. However, it is crucial to highlight the complexity of herbal tea compositions and their multiple constituents; therefore, caregivers may use remedies recommended by family and friends and find them beneficial; however, they should be cautious when using these remedies, particularly concerning their potency and recommended dosages [18]. Our study concluded that 30.2% of mothers do not use herbal medicines, and anise was the most used one, which differs from a Nigerian study that reported herbal medicines comprised 51.8% of the self-administered treatments for infants with colic [23]. Among pharmacological options, simethicone was the mothers' most frequently employed drug in the present study, followed by probiotics. The efficacy of both these medications was validated through clinical approval by a significant number of clinicians, as established in a Turkish study that recommended it as an exclusive medical intervention for treating IC, with observed clinical benefits [24].

Our findings show that most mothers (56.5%) received information about IC from various sources, including healthcare professionals, neighbors, family members, and husbands, and only 27.7% of them exclusively relied on healthcare professionals. Moreover, only 3.9% of mothers mentioned consulting a doctor for managing colic episodes; in comparison, 48.8% learned about IC management strategies through diverse sources such as healthcare personnel, the internet, social media, and television.

Limitations

This study's cross-sectional design with a sample size of 285 participants from a single region, Jazan, might only partially represent the overall view of IC across Saudi Arabia; it offers associations but only allows for establishing causality or observing changes over time. Our study relies on questionnaire responses containing limitations such as response variability, reaching only mothers who have access and can use the internet, understanding disparities, false answers, and recall bias; thus, longitudinal studies are recommended to provide accurate results about mothers' knowledge and attitudes about IC and its management practices.

## Conclusions

This study explored that most mothers did not get information about IC from healthcare staff and did not agree to consult a doctor to guide its management. These findings emphasize the necessity for comprehensive educational support strategies for expanding awareness about self-diagnosis and encouraging medical consultation and management of IC that should be initiated at the antenatal care visits and continued throughout the routine postnatal well-baby clinic to alleviate the discomfort of mothers and infants.
